# 

**Published:** 1855-09

**Authors:** 


### INDEX TO VOLUME I.

PAGE.

A Case of Parturition, with its complications, &c. 11
An Oleaginous Element Indispensable in Pthisis. 28
A Case of Hysteria, complicated with Mania-a-Portu 182
A Case of Violent Uterine Hemorrhage, occurring twelve days after Delivery. 202
An Essay on Pneumonia 321
An Essay on Quackery 513
Abnormal Placenta 518
A Case of Partial Placenta Prævia, complicated with transverse presentation and rigidity of the Os Uteri 591
Anæsthetics in the Austrian Army 448
A great Medical Discovery 49
A new method of Lithotomy in Women 125
A very Creditable Case 52
Acid Beef-Tea 384
A New Operation for Lacerated Perinæum. 118
A Case of Symphyseotomy 507
Alleged Cure of Cancer 116
Account of a Case of Catalepsy, &c. 166
Abortion following the Administration of Chloroform 348
Additional Observations on the Invariable Existence of a Premonitory Diarrhœa in Cholera 404
Average Duration of Human Life 408
An Overdose of Homœopathy 489
Attempt at Self-Castration 490
An Unique Case of Dislocation of the Patella 620
Annual Address of Prof. George B. Wood 670
A Chapter of Accidents 683
An Honest Quack 255
Atlanta Medical College 56-243-433-630-686
Alexander Means, M. D. 63
Atlanta Medical Society 372
American Medical Association 318
Abstract of the Proceedings of the Ninth Annual Session of the American Medical Association 686-691
An Address, Introductory to the Second Course of Lectures in the Atlanta Medical College 705
A Thesis on Typhoid Fever, presented to the Faculty of the Oglethorpe Medical College, at Savannah, Ga. 723
Bristol (Mass.) South District Medical Society 242
British Association for the Advancement of Medical Science 447
Bibliographical 246-309-437-496-632-688
Blister & Critic 248
Bunion 62
Book Notice 63
Cholera 269
Case of Nephritic Colic 33
Chloroform—Cholera 64
Considerations on the Reciprocal Influence of the Physical Organization and Mental Manifestations 157-533
Clinical Lectures on Hypertrophy of the Prostrate 215
Cultivation of Liquorice in this Country 511
Case of Lodgement of Foreign Body in Air-Passages 231
Case of Yellow Fever occurring in St. Louis. 289
Caustic Pulverized 383
Case in which Six Drachms of Arsenic were taken 305
Chloroform in Labor 499
Chemical Pathology 305
Circular 505
Chemical Lecture at St. John's Hotel for Invalids 351
Card of the Committee on Prize Essays 382
Case of Triplets, &c. 491
Case of Encysted Sanguineous Tumor of the Neck 557

PAGE.

Chloroform in Pneumonia 447
Close of the Volume 757
Diabetes treated by Fermented Barley 254
Dislocations of the Clavical 236
"Discretion is the better part of Valor" 307
Description of a Case of Frontal Pain relieved by large doses of Hydriodate of Potassa 472
Death of M. Berger 448
Druggists, Pure Medicines, &c. 122
Dr. Garland's Letter 315
Death from Chloroform 377
Death of a Fœtus from Smallpox 575
Dislocation of the Knee Joint, with Fracture of the Patella 17
Difficulty and Importance of Correct Diagnosis, and especially in certain Nervous Affections 455
Dry Gangrene 764
Essay on Croup, read before the Atlanta Medical Society 385
Extract from the Records of the Atlanta Medical Society 400-460-524
External Employment of Oil of Turpentine in Consolidated Lungs 380
Extraction of a Uterine Pessary from the Bladder 127
External Use of the Acid Nitrate of Mercury 51
Exchanges 191
Excision of the Cervix Uteri for Corroding Ulcers 483
Expulsion of Tænia by Pumpkin Seeds 446
Epidemical and Local Influences on Diseases 493
Errata 320,691,764
Fluid Extracts 128
Fibrinous Bodies in the Heart after Death 355
Food and its Adulterations 89
Formulæ for the Internal Administration of Chloroform 508
Foreign 256
Fleming's Hygienic Journal 61
Glycogenia—The secretion of Sugar, in the Human Economy 281
Heavy Damages for Slander of a Physician 128
Honor to whom Honor is due 185
Hospital Reports—Hysterical Affection of Hip Joint 222
Hypnotism in Hysterical Paralysis 427
Hydrocele 126
Iodine in Fibrous Tumors of the Uterus 124
Inhalation of the Fumes of Opium in Coryza 128
Inoculation in Yellow Fever 97
Illustrations of the Influence of Pregnancy. 294
Indiscriminate Drugging 443
Intense Cold as a Local Anæsthetic 20
Insanity in Relation to Crimes 601
Jaundice 256
Longevity of the Negro 254
Lectures on Clinical Surgery 109
Lard as an Antidote to Strychnia 382
Law Cases bearing upon the subject of Insanity 412
Letter from Paris 468
Legal Responsibility 576
Medical Jurisprudence—Prolonged Retention of Life by Infants who have not breathed 38
Medical College of Virginia 256
Manual of Clinical Medicine, &c. 123
Medical Journals—Should the Practitioner read them? 379
Medicine As It Is 1
Medical Reform 369
Males and Females 252
Medical Periodicals 54
Mt. Syme on Chloroform 54
Malaria and its Cause and Effects 218
Mt. Valleix 126-241

PAGE.
Malaria not the cause of Fever................ 342
Museum, Atlanta Medical Men, &c............ 565
Medical Affairs in Europe........................ 575
Memoir on the Simple Ulcer of the Stomach........................................... 615
Medical Inquiries connected with our Minister to France................................. 250
Medical Etiquette and Professional Rights.. 759
New Chemical and Philosophical Apparatus 80
Napor of Iodine in Mammary Tumors....... 53
Note on the Induction of Sleep and Anæsthesia by Compression of the Carotids..... 117
New Orleans School of Medicine............... 691
Newspaper Recommendations of Quack Medicines.......................................... 319
New French Works.................................. 253
New Remedies........................................ 381
Our Enterprize........................................ 183
Omission................................................ 192
Observations on the Rigidity of the Os Uteri and Perinæum.................................. 477
Observations on Curative Evidence........... 290
On Diseases of Infancy............................ 30
On Sterility depending on certain diseased states of the Lining Membrane of the Womb—Its Treatment and Cure............ 152
On Uterine Pains and Hemorrhage after Delivery........................................... 173
On the Treatment of Cholera with Strychnia........................................................ 180
On the use of Sulphate of Cinchonia as a Substitute for the Sulphate of Quinia...... 274
On the Rudimentary Reproduction of Extremities after their Spontaneous Amputation........................................... 296
On Intra-Uterine Goitre, or Bronchocele..... 298
On the Variation of Mothers' and Nurses' Milk.................................................. 255
On Chronic Organic Liver Affections.......... 70
Obstinate Vomiting in Pregnancy, with Abortion.............................................. 75
Operation of Tracheotomy in Croup.......... 459
Our Journal............................................ 119
Oglethorpe Medical College..................... 247
On some of the Local and Remote Effects of Chloroform, hitherto unnoticed.......... 419
On Varioloid and Varicella........................ 479
On the Prevention of Pitting in Smallpox... 481
On the Contagiousness of Hooping Cough... 506
On the destruction of Non-Vascular Nœir by the Vienna Caustic.......................... 509
On the presentation of Dead Children........ 574
On the Practical Application of Chloroform as a Topical Anæsthetic to Mucous and Cutaneous Surfaces.............................. 610
Observations on the Cause of the Disease known as the Sun-Stroke..................... 752
Paralysis suffering after Anæsthesia.......... 199
Pharmacy in Baltimore............................ 510
Prolapsus Uteri....................................... 233
Prosecution of Malpractice........................ 224
Progress of Discovery in the Nervous System, &c................................................ 529
Probable Fracture of Base, or Scull, from a fall....................................................... 680
Prize Questions........................................ 248
Premature Labor...................................... 192
Pathology of Fevers................................. 65
Physiological and Pathological Observations connected with the effects of Alcoholic Drinks upon the Liver.................... 133
Paracentesis Thoracis, Translated from the French................................................. 137
Peculiarities and Diversified Symptoms Manifested in Hysterical Subjects........... 520
Prof. Austin Flint, M. D........................... 124
Publications Received.............................557-762
Quick Progress of Mercurial Ointment....... 512
Report of the Committee on Scarlatina..... 360
Report on Cases of Fever......................... 421
Report of a Committee on Home Adulterations............................................... 475
Remarks on the Diagnosis of Dislocations of Shoulder Joint................................ 465
Rheumatic Pneumonia............................. 245

PAGE
Rupture of the Heart................................ 188
Rheumatism........................................... 64
Reports from the Case-Book of J. Junius Harris, M. D., and Horatio N. Hollifield, M. D., of Tennille, Georgia...................... 21
Report of the Surgical Clinic of the Atlanta Medical College..........................24-86-129-204-329
Report of Two Surgical Cases................... 208
Remarks on the Pathology of Phlegmasia Dolus................................................ 257
Reports from the Case-Book of Thomas S. Powell, M. D., of Sparta, Georgia.......15-264-516
Remarkable Case of Tumor of the Side...... 272
Report of a Case of Shoulder Presentation 744
Secretion of Milk, excited by Nursing, in the Case of a Grandmother.................... 65
Stramonium as a Remedy in Dysmenorrhœa................................................ 392
Smallpox as it appeared in Richmond, Virginia, and its vicinity in the Winter of 1855-6............................................... 577
Salivary Calculus.................................... 594
Salutatory.............................................. 55
Serile Insanity......................................... 363
Stramonium in Dysmenorrhœa................. 445
Subscriptions Received.................484-448-576-691
Successful Treatment of Traumatic Tetanus..................................................... 416
State Medical Society..............................506-564
Solution of Tannin in Aromatic Sulphuric Acid as a Hœmostatic........................... 383
Simple and Puerperal Peritonitis.............. 624
Sugar and Salt for Chronic Congestion of the Cervix Uteri.................................. 128
Subscribers, Contributors, &c.................... 246
Three Surgical Cases............................... 35
The Pathology and Treatment of Leucorrhœa................................................... 40
The Causes of Hemorrhage from the Unimpregnated Uterus.............................. 146
Tracheotomy for the removal of a Foreign Body.................................................. 449
The Diseases &c, of the Bible.................... 641
The Medical Virtues of the Compound Fluid Extract of Tephrosia Virginiana............ 77
Thesis on the Sign of Pregnancy, presented to the Faculty of the Atlanta Medical College..................................................... 333
The Monthly Stethescope and Medical Reporter................................................ 504
Tracheotomy in Childhood........................ 507
The Last Phase of Quackery..................... 512
Treatment of Croup................................. 541
Tuberculosis........................................... 553
The Second Session................................ 562
To Contributors...................................... 631
Translations from Foreign Journals.......... 678
To Book Publishers................................. 448
Treatment of Sciatica.............................. 249
The Value of Instinct in the Choice of Diet 251
" The Noble Army of Martyrs".................. 253
Tape Worm Expelled by Infusion of Pumpkin Seed............................................. 125
Twins born at an Interval of Forty Days... 126
The Inadvisability of Closing Wound after Operation for Hernia.......................... 127
Tic Doloureux......................................... 64
The Cincinnati Medical Observer.............. 372
Tilden & Company's Extracts................... 373
To Correspondents.................................. 384
To Our Friends........................................ 306
The State Lunatic Asylum........................ 320
The Musk-Deer....................................... 748
To those whom it may concern................. 764
To Subscribers........................................ 759
Uterine Pains and Hemorrhage after Delivery................................................. 103
Uterine Displacements............................ 127
" Vis Medicatrix Naturæ and Autistasis"... 193
Vapor of Iodine in Treatment of Ophthalmia..................................................... 221
Vicarious Menstruation........................... 211
Viratrum Veride..................................... 666
Vitis Vinifera Radix, as a Diuretic............ 746
Yellow Fever.......................................... 120

ANNUAL

### ANNOUNCEMENT OF LECTURES

IN THE

### Atlanta Medical College,

FOR THE

### SESSION OF 1856,

WITH CATALOGUES OF THE

### Students and Graduates.

ATLANTA, GA.:
PRINTED BY C. R. HANLEITER AND CO.,
1855.

### BOARD OF TRUSTEES.

PRESIDENT OF THE BOARD,

DR. JOSEPH THOMPSON.

SECRETARY,

JOHN COLLIER, ESQ.

GREEN B. HAYGOOD, ESQ.,

JARED IRWIN WHITAKER, ESQ.,

HON. WILLIAM EZZARD,

JOHN L. HARRIS, ESQ.,

LEONARD C. SIMPSON, ESQ.,

T. M. DARNALL, M. D.,

WILLIAM HERRING, ESQ.

### FACULTY.

H. W. BROWN, M. D.,
*Professor of Anatomy.*

J. W. JONES, M. D.,

*Profeseor of Principles and Practice of Medicine.*

ALEXANDER MEANS, M. D.,
*Professor of Chemistry and Pharmacy.*

W. F. WESTMORELAND, M. D.,
*Professor of Principles and Practice of Surgery.*

JESSE BORING, M. D.,
*Professor of Obstetrics and Diseases of Women and Children.*

JOSEPH P. LOGAN, M. D.,
*Professor of Physiology and General Pathology.*

J. G. WESTMORELAND, M. D.,
*Professor of Materia Medica and Medical Jurisprudence, and Dean of the Faculty.*

S. W. ANTHONY, M. D.,
*Demonstrator of Anatomy.*

J. G. McLIN,
*Janitor.*

### ANNOUNCEMENT.

The Second Course of Lectures in this Institution will commence on the first day of May next, and close the last of the following August.

The sentiment expressed in the first announcement, that "a Summer course of Lectures" was called for in the South, met a favorable response from a respectable number of Students, even under the most unfavorable circumstances imaginable.

Considering the great pecuniary pressure; the shortness of the time, and imperfect manner in which notice was given, and the unfavorable impressions that were made toward the Institution in various parts of the country, it was indeed encouraging to witness the number and character of those who composed the first class in the Atlanta Medical College.

### Apparatus, Preparations, &c.

A sufficient quantity of Anatomical and Surgical preparations will be procured before the commencement of the next Session to afford additional facilities for the study of Anatomy and Surgery during the next Course of Lectures.

Large plates, exhibiting the blood vessels, muscles, &c., in all the important surgical regions, are already in possession of the Professor of Surgery.

Important additions to the Chemical Apparatus will be made by the Professor recently elected to the chair of Chemistry, who will have manufactured to order, and in person make selections of such articles as his long experience in practical Chemistry has proved most useful in a Laboratory. Professor Means will give the necessary instructions to his manufacturer in Philadelphia, in time to be prepared for commencing his course with a new and splendid apparatus, containing such late improvements, by himself and others, as he considers valuable.

The Professor of Physiology will be abundantly supplied with plates, models, &c., for the proper elucidation of his department of science.

The Professor of Obstetrics, already provided with preparations, manakin, &c., will have in addition plates and an ample collection of wet and dried preparations, suited specially to the study of Midwifery and diseases peculiar to females.

In a word, each department will be provided with the necessary means to facilitate the pupil in acquiring a thorough medical education.

### Clinical Instruction.

By reference to the reports of the Medical and Surgical Clinic that will appear in the Atlanta Medical and Surgical Journal, it will be seen that the number and importance of the cases during the last course were sufficient to afford considerable practical instruction to the class. By the increased number of patients in an Infirmary, belonging to two members of the Faculty, and by the assiduous exertions of each Professor, they hope to afford still greater facilities for Clinical instruction, which will be given, as heretofore, twice a week during the course.

### Practical Anatomy.

During the past course of Lectures it has been satisfactorily proven that preserved subjects for the purposes of dissection by the student, and for Anatomical demonstrations, are equal to fresh material, and in point of comfort to the demonstrator or dissector, far excel them even in the *winters* of our climate.

Arrangements will be effected to have put up during the winter a sufficient number of such to answer for the study of Practical Anatomy during the whole course, so that the putressence occasionally found in those prepared after the warm season commences, may be avoided entirely.

The Anatomical rooms, thus supplied with material, will be opened by the 20th of April for those who may wish instruction in the art of dissecting and to pursue the study of Practical Anatomy.

### College Building.

The College Edifice, the corner stone of which was laid in July last, is now in process of erection, and the work, which is steadily progressing, will be sufficiently advanced to afford Lecture rooms for the next session. The ground plan is ninefive by sixty-five feet, divided by three partition walls, so as to make four rooms with a hall of 13 feet running through the centre of the house. The building will be two stories high, exclusive of the basement, and will contain eight rooms including three large lecture rooms. The Amphitheatre and dissecting room will be provided with sky-lights.

### Requisites for Graduation.

In order to be admitted to examination for the degree of Doctor of Medicine the student must be twenty-one years old and of good moral character. He must have attended two full courses of lectures in a respectable school of medicine, the last of which must be in this Institution.

Three years' study under the direction of a competent instructor in medicine will be required, including, of course, the terms of lectures.

An original Thesis on some medical subject, of the student's own selection, must be presented to the Dean of the Faculty one month before the close of the Lectures.

### Fees for the Course.

| | |
|---|---|
| FULL COURSE OF LECTURES, | $105 |
| MATRICULATION FEE (payable once only), | 5 |
| DISSECTING TICKET (taken once only), | 10 |
| GRADUATION FEE, | 25 |

Board which, from the scarcity of provisions during the past Summer, commanded from three and a-half to four dollars per week, will be reduced at least one dollar by the abundant crop of provisions of every description.

### CATALOGUE OF STUDENTS.

| *Names.* | *Residence.* | *Preceptor.* |
|---|---|---|
| WM H BOYD | Erin, Ga | J G and W F Westmoreland. |
| MARTIN G SLAUGHTER | Pinckneyville, Ala | J W Jones. |
| DANIEL R LEECH | Charlotte, Tenn | J F Davis. |
| JOHN O HOLLOWAY | Waynansville, Ga | David Kendall. |
| A M PARKER | Atlanta, " | Boring & Parker. |
| A P CANNON | Fayetteville, " | W H Blalock. |
| R J WESTMORELAND | Whitewater, " | Joseph A McGehee. |
| J B MANGHAM | Acworth, " | Burnsides & Smith. |
| A W GRIGGS, M D | Newnan, " | University of Nashville. |
| J J JORSEY | Spring Hill, Ala | J G & W F Westmoreland. |
| W M BRAY | Atlanta, Ga | " " |
| A B WALLACE | Lawton, " | D S Perkins. |
| A C JONES | Atlanta, " | T M Darnall. |
| G W HUMPHRIES | " " | Gilbert & Wilson. |
| R M NEWTON | " " | Wm B Thomason. |
| W H WILLBANKS | Mt. Hickory, " | S L & J M Hamilton. |
| S G SANDERS | " " | " " |
| W P PARKER, M D | Atlanta, " | Medical College of Georgia. |
| ELI FROST | Marietta, " | Practitioner. |
| J J CALDWELL | Zebulon, " | " |
| W T C CAMPBELL | Atlanta, " | J G & W F Westmoreland. |
| B F DARNALL | Roanoake, Ala | J H Davis. |
| E J ROACH, M D | Atlanta, " | University of Maryland. |
| B O JONES | " " | Practitioner. |
| THOS T KEY | " " | T M Darnall. |
| B F BOMAR | " " | Practitioner. |
| THOS BORING | " " | " |
| JOHN J BORING | " " | Boring and Parker. |
| C R MOORE | Spier's T. O. " | William Houser. |
| M J ROBINSON | Millen, " | F W B Perkins. |
| M H OLIVER, M D | Atlanta, " | University of Nashville |
| THOS CULL | Washington, " | G W Palmer. |
| J H HARRIS | Auburn, Ala | J W Jones. |
| T M DARNALL | Atlanta, Ga | Practitioner. |
| L H JORDAN | Colaparchee, " | J J Caldwell. |
| N B DREWRY | Erin, " | William Westmoreland. |
| N L ANGIER | Atlanta, " | Practitioner. |
| L L LEDBETTER | " " | Thomas M Darnall. |
| J H PRICE | Washington, " | James W. Price. |
| J M WILLIAMS | Ringgold, " | Clements and Russell. |
| G L KEY | Jackson, " | J. M. McMichael. |
| G W T STAMPS | Bowden, " | Practitioner. |
| G M SCARLETT | Brunswick, " | Howard & Johnston. |
| W H PHILPOT | Auburn, Ala | John J Mason. |
| W H RYALS | Decatur, Ga | P F. Hoyle. |
| T P BURGAMY | Griffin, " | M C Purifoy. |
| G B WEEDON | St. Augustine, Fla | J Washington Jones. |
| J H JONES | Eagleville, Tenn | S S McLemore. |
| J BIVENS, M. D | McDonough, Ga | University of Nashville. |
| CHAS CUMMING | Atlanta, " | Practitioner. |
| J M HAMILTON | Mt. Hickory, " | Practitioner. |
| B GARRETH | " " | Hamilton & Brother. |
| W T BARRON | Adairsville, " | Bomar & Layton. |
| R B BADGER | Atlanta, " | T M Darnall. |
| A TILDON | Cussetta, Ala | John W Jones. |
| W O HANSON | St. Cloud, Ga | W C Redwine. |

| *Names.* | *Residence.* | | *Preceptor.* |
|---|---|---|---|
| Wm S Barrett, | Walnut Grove, | Ga | Practitioner. |
| J D Keaton, | Newton, | “ | G E Pryor. |
| J W Farill | Lithonia, | “ | W. P. Bond. |
| E Griffin, | Atlanta, | “ | J G & W F Westmoreland. |
| G W Neely, | Fairburn, | “ | A. R Welborn. |
| N F Powers, | Atlanta, | “ | Practitioner. |
| C S Haley, | Hebron, | “ | John R Tucker. |
| J J Newsome, | Sandersville, | “ | J R Smith. |
| R M Waldrop, | Rocky Mount, | “ | F M Brantly. |
| C P Brown, | Madison, | “ | H J Ogilby. |
| W A Gillespie, M. D. | Jonesboro, | “ | Jefferson College. |
| W L Starnes, | Conyers, | “ | Robert A Johnson. |
| J E G Terrell, | Greenville, | “ | J W Anthony. |
| T J Bailey, | Jackson, | “ | J W Jones. |
| P M Tidwell, | Fairburn, | “ | W P Ragland. |
| S Biggers, | Atlanta, | “ | Practitioner. |
| J W Wofford, | Clarkesville, | “ | A P Houston. |
| W H Blalock, | Fayetteville, | “ | B O Jones. |
| D B Juhan, | Stone Mountain, | “ | G K & J L Hamilton. |
| J A Johnson, | Mt. Hickory, | “ | J M & S L Hamilton. |
| W A Culbertson, | Cave Spring, | “ | J N Culbertson. |
| J W. Bozeman, | Dallas, | “ | J M Bledsoe. |

### Graduates.

| *Names:* | *Residence.* | *Subject of Thesis.* |
|---|---|---|
| M G Slaughter, | Alabama | Inflamm'n & Ulceration Cervix Uteri. |
| J E G Terrell, | Georgia | Puerperal Peritonitis. |
| N B Drewry | “ | Arterial System. |
| C S Haley, | “ | Diagnosis Treatment and Prognosis. |
| A B Wallace, | “ | Acute Dysentery. |
| W H Willbanks, | “ | Puerperal Fever. |
| J A Johnson, | “ | Intermittent Fever. |
| S G Sanders, | “ | |
| R M Waldrop, | “ | Remittent Fever. |
| C R Moore, | “ | Typhoid Fever. |
| B O Jones, | “ | Tephrosia Virginiana. |
| J H Jones | “ | Epidemic Cholera. |
| G W Neely, | “ | |
| G W T Stamps, | “ | Typhoid Fever. |
| W O Hanson, | “ | Fractures. |
| J J Newsome, | “ | Signs of Pregnancy. |
| G M Scarlett, | “ | Intermittent Fever. |
| Thos Cull, | “ | Scarlatina. |
| P M Tidwell, | “ | Menstruation. |
| J D Keaton, | “ | Rheumatic State of Fever. |
| G W Humphries, | “ | Acute Gastritis. |
| D B Juhan, | “ | |
| T. P. Burgamy | “ | Pneumonia. |
| J M Hamilton, | “ | Menstruation. |
| Thos Boring, | “ | Congestive Fever. |
| Eli Frost, | “ | Puerperal Fever. |
| C P Brown, | “ | Pneumonia. |
| B F Bomar, | “ | Typhoid Fever. |
| W A Culbertson, | “ | Infantile Remittent Fever. |
| T M Darnall, | “ | Scarlatina. |
| D R Leech, | “ | Scarlatina. |
| L L Ledbetter | “ | First Dentition. |

The following gentleman were admitted *ad eundem gradum* in this Institution:

W. P. Parker, M. D., and M. H. Oliver, M. D., Georgia.

The Honorary Degree was conferred on Wm. T. Feay, Savannah, Georgia.

*Medical Jurisprudence.*—Prolonged Retention of Life by Infants who have not breathed........ 38

The Pathology and Treatment of Leucorrhœa........ 40

A great Medical Discovery—Mercury taken from the system by Electricity.... 49

External Use of the Acid Nitrate of Mercury........ 51

A very Creditable Case—Life saved by the Doctor........ 52

Napor of Iodine in Mammary Tumors........ 53

Mr. Syme on Chloroform........ 54

Medical Periodicals........ 54

Editorial and Miscellaneous........55

☞ Mr. A. TILDEN is an authorized Agent for this Journal.

**PRIVATE MEDICAL INSTRUCTION.**

THE undersigned design to give a Course of Private Instruction to such Medical Students as may desire to enter their office. Regular lectures and examinations on the various branches of medical science will be afforded twice a week.

J. G WESTMORELAND, M. D.,
ATLANTA, Sept. 1, 1855. W. F. WESTMORELAND, M. D.

